# Capturing Subjective Age, Subjective Life Expectancy, and Their Links With Older Adults’ Health: The Dutch Longitudinal Aging Study Amsterdam

**DOI:** 10.1177/08982643211004001

**Published:** 2021-03-31

**Authors:** Dorly J. H. Deeg, Erik J. Timmermans, Almar A. L. Kok

**Affiliations:** 1 Amsterdam Public Health Research Institute, Epidemiology and Data Science, 1209 Amsterdam UMC Vrije Universiteit Amsterdam, Amsterdam, The Netherlands; 2 Amsterdam Public Health Research Institute, Psychiatry, 1209 Amsterdam UMC Vrije Universiteit Amsterdam, Amsterdam, The Netherlands

**Keywords:** subjective lifetime perspectives, graphical measure, numerical measure, physical health, mental health

## Abstract

**Objectives:** This study compares the associations of two subjective lifetime perspectives, subjective age (SA) and subjective life expectancy (SLE), with physical performance, self-rated health, and depressive symptoms. **Methods:** 64 91-year-old participants were selected from three waves of the Longitudinal Aging Study Amsterdam (2008/09, 2011/12, 2015/16; *n* = 1822 participants, *n* = 3500 observations) that included graphical and numerical measures of SA and SLE. We used generalized estimating equations to examine their associations with health. **Results:** Associations of SA/SLE with health were weaker for physical performance than for self-rated health and depressive symptoms. The associations of SA and SLE with physical performance were of similar magnitude but with self-rated health depended on the type of measure. Depressive symptoms, instead, showed a stronger association with SA than with SLE. Graphical measures showed weaker associations than numerical measures. **Discussion:** The way in which subjective lifetime perspectives and health are conceptualized and measured influences the strength of their associations.

## Introduction

As people pass through successive life stages, their perception of their position in their lifetime develops (Barrett & Montepare, 2015). This perception can be expressed in two ways. The first is how old one feels; the second is how much time one feels one has left to live. These two opposite time perspectives are commonly referred to as subjective age (SA) and subjective life expectancy (SLE), respectively. Both shape people’s perception of the place they currently occupy in their lifetime. In the following, we use the term “subjective lifetime perspectives” as an umbrella term for SA and SLE.

Several studies have shown that the longer the older people’s SLE, the more likely they are to engage in activities such as paid work and volunteering and in healthy behavior (Adams et al., 2015; Lee et al., 2019; Van Solinge & Henkens, 2010). Moreover, SLE has been shown to predict actual survival time (Van Solinge & Henkens, 2018). Likewise, evidence shows that the younger the people’s SA, the greater their work engagement, the better their (mental) health, and the longer their survival (Keyes & Westerhof, 2012; Uotinen et al., 2005; Ye & Post, 2019). The evidence of objective consequences of subjective lifetime perspectives makes these of interest to a wide range of disciplines, as well as to economic policy, public health, social work, and personal life course planning.

### Concepts Related to the Two Subjective Lifetime Perspectives

Subjective age is considered to be one indicator of subjective perceptions of aging (Hausknecht et al., 2020). It is closely linked to the life stage in which individuals perceive themselves to be and depends on what an individual believes a certain age is meant to feel like. As Barrett and Montepare (2015) state in their review of SA, we live in an age-differentiated world in which certain social roles and behaviors are viewed to fit to certain chronological age categories. This social structuring of the life course feeds into “internalized conceptions of the life course that include beliefs and expectations about past, present, and future age-related behaviors and events” (p. 58). Against these conceptions, individuals evaluate their developmental movement and position in their lifetime. Thus, the timing and the number of transitions individuals experience as they age, for example, children leaving home, onset of disease, or retirement, and the gains and losses that they imply, affect how old they are likely to feel. Social expectations play a distinct role in this process. This is why SA is also regarded as a reflection of internalized age stereotypes (Diehl et al., 2015; Stephan et al., 2015).

Another line of argumentation is that as individuals reach older age, they attempt to maintain continuity in their self-identity by feeling younger than their chronological age (Westerhof et al., 2012). This basically cognitive process is driven by self-enhancement: the desire to maintain or improve one’s self-esteem. In turn, the drive of self-enhancement is dependent on the sociocultural context and has been shown to be greater in individualistic cultures than in cultures that are based on solidarity. To illustrate, Liang (2020) compared the SA of urban and rural older Chinese. The latter, who highly valued interdependence, felt older than their urban counterparts, who highly valued independence. Moreover, feeling old predicted depressed mood in urban older Chinese but did not in their rural counterparts. The author concluded that maintaining a youthful age identity proved to be a useful strategy for self-enhancement or self-protection only in an individualistic culture. The current study is based in the Netherlands, a welfare state that is traditionally based on solidarity (Westerhof et al., 2012).

Regarding SLE, at some point in their lifetime, individuals start being aware of the finitude of their life (Neugarten, 1979). They realize that they have lived more years in the past than they will live in the future and start perceiving the number of years they have left to live as limited. Triggers of this awareness of finitude may in part derive from the number of transitions experienced during the life course, bodily signs of poor health, or approaching the age at which a parent died (Zick et al., 2014). For another part, perceiving future life as limited may originate from the realization that one has not fulfilled all ambitions that one coveted. The awareness of finitude may help to weed out less important ambitions and focus on one’s remaining ambitions (Diehl et al., 2015).

Considering their distinct conceptual connotations, the two subjective lifetime perspectives implied by SA and SLE are not necessarily closely linked. Empirically, in two studies that included both perspectives, their correlations ranged from only −.26 to −.14 (Palgi, 2016; Shrira et al., 2014). Feeling younger may not mean that an individual perceives a longer life expectancy, and perceiving a shorter life expectancy may not mean that an individual feels older. Therefore, it makes sense to examine both time perspectives as complementary aspects of subjective lifetime.

### Subjective Lifetime Perspectives and Health

Ample evidence shows a mutual relationship between poorer physical and mental health and both older SA and shorter SLE (see e.g., meta-analyses by Debreczeni & Bailey, 2020 and Westerhof et al., 2014 and narrative reviews by Diehl et al., in press, Gabrian et al., 2017, and Wurm et al., 2017). The distinction between the concepts of SA and SLE, however, may play out in differential associations of the two subjective lifetime perspectives with health. On the one hand, as noted above, older SA is considered to reflect, among others, an increased number of transitions that individuals experienced throughout life, among which the onset of disease and disability. On the other hand, shorter SLE reflects an awareness of finitude, triggers of which may derive from bodily signs of poor health. Thus, poor health is a likely predictor of both subjective lifetime perspectives. Vice versa, feeling older may cause psychological distress and may undermine one’s sense of control with implications for health (Wurm et al., 2017), and perceiving a shorter life expectancy may cause death anxiety and may lead individuals to neglect investing in health (Gabrian et al., 2017; Palgi, 2016). Thus, both subjective lifetime perspectives are likely predictors of health. To disentangle possibly different underlying mechanisms, two empirical questions need to be addressed. First, is health more strongly associated with SA or with SLE? Second, does the differential strength of these associations depend on the aspect of health that is examined?

Before we discuss available literature pertinent to these questions, we note that the body of literature on health and SA is largely separate from that on health and SLE. Reviewing these literature bodies, we found that studies on SA were mainly published in psychology and surprisingly few in public health and social science journals, whereas studies on SLE were represented about equally in psychology, public health, and social science journals. Bringing together these two literature bodies will provide a wider basis for theory building about subjective time perspectives across the life course and the role of health therein.

The largely separate development of the two literature bodies implies that data on SA and SLE are not often combined in one study, and empirical evidence on which of them shows stronger associations with health leans on only a few studies. Among the three pertinent studies we could find, two were set in Israel and one in Croatia, and all were cross-sectional and included community-living samples aged 50 years and over (Ambrosi-Randić et al., 2018; Palgi, 2016; Shrira et al., 2014). The Croatian study showed similar predictive values for SA and SLE of self-rated physical (betas .26 and .21, respectively) and mental health (both betas .16). The Israeli study by Palgi (2016) showed correlations of both SA and SLE with physical health, self-rated health, and depression, and for each health indicator, these correlations were of similar size. In contrast, Shrira et al. (2014) found a greater predictive value for psychological distress of subjective distance-to-death (3% variance explained) than of SA (1% variance explained). Also, the covariates self-rated health and disability showed larger univariate associations with subjective distance-to-death than with SA. The sparse evidence, in conclusion, points to similar importance of SA and SLE for health and vice versa, but if a difference should exist, health might be more strongly associated with SLE than with SA.

A larger number of studies included various health aspects in examining associations with either SA or SLE so that there is potentially more empirical evidence on which health aspects show stronger and which show weaker associations with each subjective time perspective. Note, that most of these studies included health measures as covariates and did not aim to compare these health measures. We focus on community-based studies of older age-groups, that is, 50 years and over. Most frequently studied health measures include self-rated health, disability, and depressed affect or psychological distress. Together these constitute a comprehensive coverage of health, and as they tap into body or mind, they may well have differential associations with subjective lifetime perspectives.

We first summarize studies on SA that include at least two of these health measures as predictors. They used different samples of older people in Canada, Croatia, Germany, Great Britain, Israel, Norway, Sweden, and the United States (Ambrosi-Radić et al., 2018; Barrett & Gumber, 2020; Bergland et al., 2013; Bowling et al., 2005; Choi et al., 2014; Hubley & Russell, 2009; Infurna et al., 2010; Palgi, 2016; Rippon & Steptoe, 2018; Shrira et al., 2014; Spuling et al., 2019; Stephan et al., 2015). The general conclusion from these studies is that self-rated health is most strongly predictive of SA, but that there is no clear distinction between the predictive abilities of depressive symptoms and disability. Some studies show slightly stronger associations for disability (Bowling et al., 2005; Hubley & Russell, 2009; Palgi, 2016), others for psychological distress (Choi et al., 2014; Infurna et al., 2010), and again others show associations of similar size (Bergland et al., 2013; Stephan et al., 2015) or nonsignificant associations for both (Rippon & Steptoe, 2018).

Studies providing evidence of the association of at least two health measures with SLE took place among older people in England, 18 European countries jointly, and the United States (Benítez-Silva & Ni, 2008; Kobayashi et al., 2017; Palgi et al., 2019; Papachristos et al., 2020). They again show the strongest associations for self-rated health, followed by disability. Depression showed the weakest or no association. Palgi et al. (2019) asked Israeli study participants which of 13 factors they themselves rated as most important in evaluating their nearness to death; physical functioning ranked highest, while depressed mood ranked seventh.

In conclusion, regardless of the subjective lifetime and health measures used, and regardless of the study’s national setting, a remarkable congruence emerged in the rank order of the three health measures. Yet, as noted, most of the studies cited included the health measures as covariates, and their associations with subjective lifetime perspectives may be greatly affected by the presence of other covariates, in addition to differences in sampling designs, operational definitions, and analytic methods. A strict test of the association of SA and SLE with each health measure is possible only in one and the same study.

### Measurement of Subjective Lifetime Perspectives

There is a variety of ways to measure SA and SLE. In many studies, people are simply asked how old they feel they are or to what age they expect to live. The response to these questions is a number expressing an age. There are also more complex measures. For SA, for example, people are asked whether they feel younger or older than their chronological age (Hubley & Russell, 2009). For SLE, a widely used instrument is to ask people about the probability of living up to a certain age (Hurd & McGarry, 1995). The choice of particular measures may yield associations with health that differ in strength.

The great majority of the subjective lifetime measures thus ask people for a number. However, several authors note that having to come up with a concrete number may make people apprehensive, in particular when it concerns a future time perspective (Shmotkin, 1992; Teppa et al., 2015). This apprehension may lead study participants to skip such questions. Indeed, the nonresponse rate in subjective lifetime studies is substantial. In the landmark study on SLE by Mirowsky (1999), the item nonresponse was over 20%. The nonresponders were older (i.e., over 45 years), had a lower education and lower sense of control. In a study by Dormont et al. (2018), the item nonresponse was 15% and associated with lower education and poorer health. Lower educated participants, those with a weaker sense of control, and those in poorer health may feel more apprehension than their better-off counterparts. To avoid apprehension and item nonresponse, a graphical mode of administration of subjective lifetime measures may be recommendable, for example, a Cantril ladder with rungs stepping up from lowest to highest (Shmotkin, 1991) or a drawn line representing one’s lifetime (Bruine de Bruin & Carman, 2018; Demiray & Bluck, 2014; Rutt & Löckenhoff, 2016). Moreover, a graphical mode of administration may make it less likely that participants anchor their answer to their chronological age and its social connotations, as tends to be the case with numerical questions (Barrett & Montepare, 2015). Regarding SA, the common question “How old do you feel” may evoke internalized age stereotypes because the term “old” is often used as a pejorative, thus casting doubt on the validity of this question (Gendron et al., 2018). In all, graphical measures may yield more valid associations with health.

In the current study, we compare responses to numerical questions with a graphical “lifeline” from the beginning to the end of life, on which participants position themselves.

### Research Questions and Hypotheses

We compare the extent to which the two subjective lifetime perspectives, measured numerically and graphically, are associated with physical and mental health. We hypothesize, first, that younger SA and longer SLE are associated with better health, the latter association being stronger than the former, because the awareness of one’s end of life approaching may be more strongly triggered by health problems than the awareness of one’s aging (Demiray & Bluck, 2014; Shrira et al., 2014). Second, in line with the evidence described earlier, we hypothesize that associations of both SA and SLE are stronger for physical than for mental health. Third, we hypothesize that graphical measures are more strongly associated with health than numerical measures because the former are acceptable to more study participants and are measured with a minimum of context imposed and thus are likely to better represent the association with health than the latter (Bruine de Bruin & Carman, 2018).

## Methods

### Study Sample

We use data from three waves of the Longitudinal Aging Study Amsterdam (LASA, 2008/09, 2011/12, 2015/16) that include both graphical and numerical instruments to measure SA and SLE in a face-to-face interview. LASA is an ongoing longitudinal study with baseline measurement wave in 1992/93 and 3- or 4-year follow-up waves (Hoogendijk et al., 2016). Its sample was recruited from the municipal registries in 11 municipalities in three geographic regions that together represent the sociocultural variety in the Netherlands: the Protestant North-East, the Roman Catholic South, and the secularized West. The sample’s initial ages were 55–85 years, with increased oversampling of higher ages and of men. The baseline sample size was 3107. In 2003 and 2013, new samples aged 55–64 years were recruited from the same sampling frames as in 1992, with sample sizes 1002 and 1023, respectively. At follow-up measurements, these cohorts were merged with the earlier cohorts.

From each of the three waves included, participants were selected within the same age range. As the minimum age was 64 years in 2008/09, this was the lower age limit. The upper age limit was set at 91, as above this age too few participants remained. This restriction resulted in inclusion of 1222, 1180, and 1232 participants in 2008/09, 2011/12, and 2015/16, respectively. Those who did not fulfill the age criteria of 64–91 years at one or two waves or who dropped out during the study period contributed to less than three waves. Across participants, the average number of measurement waves was 2.0. Pooling the data across waves resulted in 3634 observations from 1822 participants. Among these, .7% had not filled in the lifeline. The numerical questions were asked after the respondents filled in the lifeline. On SA, 3.6% of responses were missing, and on SLE, 21.7%. On the key health measures, the nonresponse across waves was low, with missing values amounting to an additional 1.6% for physical performance and none for self-rated health and depressive symptoms.

The analytical models included, depending on the combination of health and lifetime measures, a minimum of 2732 (physical performance and numerical SLE) and a maximum of 3525 observations (self-rated health and graphical SA).

### Measures

#### Graphical lifetime measures

A “lifeline” was presented to the participant on a piece of paper (Thijssen et al., 2014). This is a 25 cm long, horizontal line with at the far left, the word “beginning” and at the far right, the word “end.” Participants were instructed that this line represented their lifespan and were asked to indicate by a cross at which point on this line they felt they currently stood. After completion of the interviews, a research assistant measured the distance (in mm) from the left end to the cross and assigned a corresponding number between 0 (beginning of the line) and 1 (end of the line). Following Thijssen et al. (2014), values below .39 were not deemed valid for persons in the age-group studied, and thus observations lower than .39 were excluded (*n* = 95, 2.6%).

From the lifeline, SA and SE are derived separately, in order to obtain constructs as close as possible to the numerically measured SA and SLE (Thijssen et al., 2014). The calculation of SA using the lifeline (LL-SA) involved, first, estimating the total lifespan by adding the participant’s chronological age and the sex-based actuarial life expectancy from the participant’s chronological age onward. Second, the score on the lifeline was multiplied with the estimated total lifespan. The calculation of SLE using the lifeline (LL-SLE) also involved two steps. First, the participant’s chronological age was divided by the value measured on the lifeline, which yielded the total lifespan perceived by each participant. Subsequently, the participant’s chronological age was subtracted from the total perceived lifespan, yielding each participant’s remaining SLE.

#### Numerical lifetime measures

The numerical questions first asked about SA, preceded by an introduction: “The next questions are about your age. People often say that they feel older or younger than they really are. We would like to know how you feel about your age. How old do you feel?” The question about SLE was phrased as follows: “How old do you think you will become?” In order to obtain remaining SLE, the participant’s chronological age was subtracted.

Previous studies on SA often report the difference between chronological age and SA, either as the absolute difference or as the proportional difference, for which the difference is divided by chronological age. As sensitivity analyses, we report our main findings also for these definitions of SA.

#### Health

Health was measured using performance-based tests, self-rated health, and depressed mood.

A physical performance score was computed using a walk test and a dress test, thus combining lower- and upper-body performance. For the walk test, participants were instructed to walk 3 m, turn around, and walk 3 m back as quickly as possible; the dress test involved participants putting on a standard cardigan. The time used for each test was recorded and categorized into four quartiles after excluding extreme values (walk test >60 seconds, dress test >80 seconds) so that the first (fastest) quartile was assigned the score 3 and the last (slowest) quartile the score 0 (Penninx et al., 2000). Summing both scores yielded a total score range of 0–6, with higher scores representing better performance.

Self-rated health was measured using the question: “How is your health in general,” with scores from 1: very good to 5: poor.

Depressive symptoms were ascertained using the Dutch translation of the 20-item Center for Epidemiologic Studies Depression Scale (CES-D, Beekman et al., 1997). Respondents were asked to indicate how often during the past week they had experienced each symptom with response categories 0: (almost) never to 3: (almost) always. The score range is 0: no symptoms to 60: maximum symptoms.

#### Covariates

Covariates included chronological age, sex, and education. Chronological age and sex are included so that in the analyses, each participant’s subjective lifetime is compared to their age peers of the same sex. Level of education has been shown previously to affect subjective lifetime perspectives as well as health (e.g., Mirowsky & Ross, 2000; Shrira et al., 2014). Age and sex were derived from population registries. Education was assessed in the interview as the highest educational level attained and recoded into number of years (5–18).

### Statistical Analyses

The data were pooled across the three waves. Because 21.6% of observations were missing on the numerical question on SLE (N-SLE), we compared their characteristics to those with valid N-SLE data. For descriptive purposes, we calculated means, *SD*s, and correlations in the pooled dataset. To assess associations between lifetime and health measures, we used generalized estimating equations (GEE; Twisk, 2003). GEE provides estimates that combine the between-subject and within-subject associations, that is, the cross-sectional association and the covariation over time of the measures of interest. To account for the interdependence of observations of individuals who participated in multiple waves, we defined an exchangeable correlation matrix. In our models, we chose health as the dependent variable because the causal relation between subjective lifetime and health has been shown to be stronger than vice versa (Spuling et al., 2013). Regardless, we examine associations, not predictive values. All models were adjusted for chronological age, sex, and wave number. A second series of models included education as an additional covariate. Associations were considered statistically significant at *p*-values < .05; for interaction terms, *p*-values <.10 were considered (Aiken et al., 1991).

The three hypotheses involve comparison of the strengths of associations across various models. This was done using the Wald statistic. In addition, for the numerical measures, it was tested what unique association of SA and SLE with health remained after including both into one model. This test was not possible for the lifeline measures, as they correlated too highly (−.91), presumably because they derive from the same instrument.

The distribution of the subjective lifetime measures showed some values that might be deemed unrealistic, that is, less than 17 for SA and over 150 for SLE. In sensitivity analyses, these were omitted from our main models.

## Results

### Characteristics of Participants with Missing Responses

Table 1 shows that participants with missing responses on the N-SLE were almost 3 years older than participants with valid responses. Correspondingly, their actuarial life expectancy was 1.5 years shorter. Their SA was 1.6 years older on the lifeline-derived measure (LL-SA) and 2.9 years older on the numerical measure (N-SA). The lifeline-derived SLE (LL-SLE) was not statistically significantly different. Regarding the health measures, the participants with missing responses had a statistically significantly lower physical performance and more depressive symptoms. There was no significant difference regarding self-rated health. Furthermore, the participants with missing responses included a relatively large share of women and persons with a lower education.

**Table 1.** Comparison of Characteristics of Respondents with Valid and Missing N-SLE data: Means and Standard Deviations, Except % for Sex.

	N-SLE Available (*n* = 2769)	N-SLE Missing (*n* = 763)	*p*-value
Age	73.6 (6.8)	76.3 (7.5)	<.001
Actuarial life expectancy	13.9 (4.7)	12.4 (5.1)	<.001
Lifeline subjective age	64.8 (10.3)	66.4 (11.8)	<.001
Numerical subjective age	64.0 (11.7)	66.9 (12.5)	<.001
Lifeline subjective life expectancy	27.9 (17.5)	28.5 (20.4)	.369
Physical performance score^a^	2.6 (1.7)	2.2 (1.7)	<.001
Self-rated health^b^	2.4 (.9)	2.4 (.8)	.194
Depressive symptoms^c^	1.8 (.9)	1.9 (.9)	.008
Sex, % females	50.6	66.8	<.001
Education in years	10.6 (3.4)	9.9 (3.4)	<.001

^a^Physical performance test score, range 0 (worst)–6 (best).

^b^Self-rated health, range 1 (very good)–5 (poor).

^c^Depressive symptoms, transformed as ln(CES-D + 1), range .0–4.1.

### Study Sample Descriptiveness

Means and *SD*s of all variables in our study, as well as their correlations, are shown in Table 2. First, we note that the numerical measures of SA and SLE correlate moderately (−.45). Second, the two SA measures show a moderate correlation (.38) and the two SLE measures show only a weak correlation (.22).

**Table 2.** Means, Standard Deviations, and Correlations Among All Study Variables (Maximum *n* = 3532).

	*M*	*SD*	Age	ALE	LL-SA	N-SA	LL-SLE	N-SLE	PP score	SRH	Depressive symptoms	Sex	Education
Age	74.2	7.0	1.0										
ALE	13.6	4.8	−.96	1.0									
LL-SA	65.2	10.7	+.50	−.50	1.0								
N-SA	64.6	11.9	+.57	−.54	+.38	1.0							
LL-SLE	28.0	18.2	−.16	+.20	−.91	−.19	1.0						
N-SLE	13.5	7.9	−.58	+.56	−.42	−.45	+.22	1.0					
PP score^a^	2.5	1.7	−.48	+.48	−.23	−.34	+.06	+.35	1.0				
SRH^b^	2.4	.9	+.14	−.13	+.12	+.18	−.06	−.30	−.27	1.0			
Depressive symptoms^c^	1.8	.9	+.19	−.14	+.11	+.21	−.03†	−.25	−.23	+.43	1.0		
Sex, % female	54	−	+.07	+.17	−.03†	+.05	+.14	−.09	+.01†	+.08	+.18	1.0	
Education^d^	10.5	3.4	−.17	+.12	+.12	−.10	−.20	+.16	+.20	−.17	−.17	−.22	1.0

*Note.* ALE = actuarial life expectancy; LL-SA = lifeline-derived subjective age; N-SA = subjective age as a number; LL-SLE = lifeline-derived subjective life expectancy; N-SLE = subjective life expectancy as number of years. ^†^Correlation coefficient is not significant (*p* > .05); all other correlations are significant (*p* < .05).

^a^Physical performance test score, range 0 (worst)–6 (best).

^b^Self-rated health, range 1 (very good)–5 (poor).

^c^Depressive symptoms, transformed as ln(CES-D + 1), range 0–4.1.

^d^Education in years of schooling.

### Association of Lifetime Measures with Health Measures

The coefficients for the numerical and the lifeline measures were generally in the same direction (Table 3, models 1). Physical performance was significantly, but weakly, negatively associated with N-SA and positively associated with N-SLE but not with either lifeline measure. Self-rated health was significantly associated with all subjective lifetime measures: the poorer the self-rating of health, the older the SA and the shorter the SLE. The largest coefficient was B = −.030 for N-SLE, indicating that a 10-year longer SLE was associated with a .3-point better self-rated health. Depressive symptoms were significantly associated with LL-SA, N-SA, and N-SLE, indicating that higher depression scores were associated with older subjective ages and with shorter subjective life expectancies. The association of depressive symptoms with LL-SLE was nonsignificant.

**Table 3.** Associations of Lifetime Measures with Health.

	Physical Performance		Self-Rated Health		Depressive Symptoms	
	Model 1	Model 2	Model 1	Model 2	Model 1	Model 2
Subjective age lifeline	+.002	−.002	+.007	+.009	+.003	+.005
	(*p* = .475)	(*p* = .395)	(*p* < .001)	(*p* < .001)	(*p* = .027)	(*p* < .001)
	Wald = .510	Wald = .725	Wald = 17.819	Wald = 31.253	Wald = 4.892	Wald = 10.509
Subjective age number	−.011	−.011	+.011	+.011	+.011	+.011
	(*p* < .001)	(*p* < .001)	(*p* < .001)	(*p* < .001)	(*p* < .001)	(*p* < .001)
	Wald = 13.868	Wald = 14.154	Wald = 27.708	Wald = 27.911	Wald = 49.001	Wald = 49.266
Subjective life expectancy lifeline	−.002	+.000	−.003	−.004	−.001	−.002
	(*p* = .203)	(*p* = .852)	(*p* = .001)	(*p* = .001)	(*p* = .197)	(*p* = .027)
	Wald = 1.608	Wald = .035	Wald = 10.126	Wald = 19.944	Wald = 1.666	Wald = 4.867
Subjective life expectancy number	+.019	+.017	−.030	−.029	−.020	−.020
	(*p* < .001)	(*p* < .001)	(*p* < .001)	(*p* < .001)	(*p* < .001)	(*p* < .001)
	Wald = 15.653	Wald = 13.553	Wald = 48.692	Wald = 47.648	Wald = 34.051	Wald = 32.919

Model 1: The data in each cell are derived from a GEE model with adjustment for age, sex, and time.

Model 2: The data in each cell are derived from a GEE model with adjustment for age, sex, time, and education.

Inclusion of education into the regression models somewhat attenuated the coefficients for the numerical measures, but they remained significant (Table 3, models 2). In contrast, the coefficients for the lifeline measures increased after including education in the models for self-rated health and depressive symptoms. Because a stronger association may indicate the presence of an interaction effect, we examined the respective interactions. In the case of self-rated health, statistically significant interaction effects were indeed present for LL-SA and LL-SLE with education (*p* = .014 and .068, respectively). In the case of depressive symptoms, the two interaction effects were not significant (*p* > .3). Models for self-rated health stratified for education show that associations with both LL-SA and LL-SLE were more than twice as strong in the higher as in the lower educated stratum (Table 4).

**Table 4.** Associations of Lifeline Measures with Self-Rated Health For Lower and Higher Educated Participants.

	Self-Rated Health	
	Lower educated	Higher educated
Subjective age lifeline	+.005	+.013
	(*p* = .023)	(*p* < .001)
	Wald = 5.206	Wald = 31.587
Subjective life expectancy lifeline	−.002	−.005
	(*p* = .051)	(*p* < .001)
	Wald = 3.821	Wald = 16.216

The data in each cell are derived from a GEE model with adjustment for age, sex, and time, with main effects, the lifetime measure, education (<10 years vs >=10 years), and the interaction term of the lifetime measure and education. The coefficients for the education strata are derived from the full model (Figueiras, et al., 1998).

The models including both N-SA and N-SLE clearly showed stronger associations of N-SLE with physical performance and self-rated health (Table 5). For self-rated health, the Wald statistic was even more than twice as high as for N-SLE compared to N-SA: 41.9 versus 17.7. Regarding depressive symptoms, instead, N-SA showed somewhat stronger associations than N-SLE: the Wald statistic was 37.2 versus 27.5.

**Table 5.** Associations of Subjective Lifetime Measures with Health: Estimates When Both are Included in One Model (Numerical Questions Only).

	Physical Performance		Self-Rated Health		Depressive Symptoms	
	Model 1	Model 2	Model 1	Model 2	Model 1	Model 2
Subjective age	−.008	−.007	+.009	+.009	+.011	+.010
	(*p* = .020)	(*p* = .020)	(*p* < .001)	(*p* < .001)	(*p* < .001)	(*p* < .001)
	Wald = 5.453	Wald = 5.431	Wald = 17.860	Wald = 17.727	Wald = 37.237	Wald = 37.205
Subjective life expectancy	+.017	+.016	−.028	−.027	−.018	−.018
	(*p* < .001)	(*p* < .001)	(*p* < .001)	(*p* < .001)	(*p* < .001)	(*p* < .001)
	Wald = 13.052	Wald = 13.052	Wald = 43.121	Wald = 41.896	Wald = 28.739	Wald = 27.528

Model 1: The data in each column are derived from a GEE model with adjustment for age, sex, and time.

Model 2: The data in each column are derived from a GEE model with adjustment for age, sex, time, and education.

### Sensitivity Analyses

The first sensitivity analysis, using for SA the difference or proportional difference with chronological age, yielded almost identical results (Supplement Table S1). The second sensitivity analysis, omitting “unrealistic” values on the subjective lifetime measures, yielded very similar results for N-SA and LL-SLE (Supplement Tables S2 and S3). For N-SLE, however, Wald statistics were greater for self-rated health and depressive symptoms, such that with depressive symptoms, the associations with N-SLE and N-SA were of similar strength.

## Discussion

In this study, we examined the associations of subjective lifetime with physical and mental health, using graphical and numerical measures of subjective lifetime perspectives. This enabled us to make a comprehensive comparison of different ways to conceptualize and measure subjective lifetime perspectives in their association with health. In contrast, most earlier studies focused only on one time perspective and a single health measure. We discuss the findings according to the hypotheses formulated.

Our first hypothesis regarding health was that SLE would show a stronger association than SA. We found both supportive and contradictive evidence. For the numerical measures, N-SLE showed stronger associations than N-SA with physical performance and self-rated health, supporting our hypothesis. In contrast, neither lifeline-derived measure was associated with physical performance, and with self-rated health LL-SA showed a stronger association than LL-SLE, most clearly in the higher education stratum, contradicting our hypothesis. For depressive symptoms, the associations of the SA measures were similar to or stronger than those of the SLE measures, whether graphical or numerical. The latter findings also contradict our hypothesis. We come back to this finding when discussing our second hypothesis.

For physical health, the awareness of one’s end of life approaching appears to be more important than the awareness of the years one has lived. However, in testing this hypothesis using the graphical measure, other forces may be at work. The measurement of SLE may be more difficult than it might seem. The apprehensiveness found in studies asking participants for a concrete number to indicate the remaining length of their lives (Shmotkin, 1992; Teppa et al., 2015) may apply to the lifeline measure as well. When confronted with the word “end” at the right-hand side of the lifeline, participants may become wary. This word might have had a similar connotation as “death.” Burke et al. (2010) use the notion of “terror management” to indicate that people, when confronted with the need to think of their own death, call up strategies that distance them from death. Such distancing would make it unlikely that study participants place a cross very near the word “end” on a lifeline. This idea is underscored by the rather weak correlation of LL-SLE with age and actuarial life expectancy (Table 2). Furthermore, thoughts about death prime a generally pessimistic psychological state, for which depressed persons may be particularly sensitive and which makes distancing a more likely behavior. This mechanism may explain why the association of SLE with depressive symptoms was weaker than that of SA.

Turning to our second hypothesis, which was based on the findings from several earlier studies, we expected that the associations of the subjective lifetime measures with depressive symptoms were weaker than with indicators of physical health. This hypothesis is not supported, as we found the weakest associations with physical performance, while the associations with self-rated health and depressive symptoms were of similar size, somewhat depending on the lifetime measure used. In particular, for SA, the association with depressive symptoms turned out to be stronger than with self-rated health. This finding may tentatively be attributed to the conceptual link of SA, and not SLE, with internalized age stereotypes and the felt need for self-enhancement (Stephan et al., 2015; Westerhof et al., 2012). Considering that our study took place in the Netherlands, a welfare state in which solidarity is relative highly valued, we would have expected that the need for self-enhancement would play a minor role and, thus, that SA and depression would show a weaker association than in more individualistic cultures such as the United States (Westerhof et al., 2012). Possibly, the value of solidarity has eroded as neoliberalism has recently pervaded many Western countries, including the Netherlands (Schrecker & Bambra, 2015). Thus, also in the Netherlands, SA may partly reflect internalized age stereotypes, and depressed older people may be particularly sensitive to these.

As a third hypothesis, we expected that measures derived from a lifeline were more strongly associated with health than numerical measures because of the presumed advantages of the lifeline. This hypothesis was not supported by the findings. In fact, the lifeline measures showed consistently weaker associations. If graphical measures indeed produce more valid associations than numerical measures, the observed weaker associations should be closer to the truth. This would imply that imposing a context using numerical measures may artificially enhance their associations with health, for example, through the social connotations attached to a specific age (Barrett & Montepare, 2015). Another possible explanation for its weaker associations is that more participants responded to the lifeline than to the numerical questions. The item nonresponders were characterized by, among others, a lower level of education, corresponding to findings from earlier studies (Dormont et al., 2018; Mirowsky, 1999). We observed that for self-rated health, associations with graphically measured SA and SLE were weaker for lower educated participants. One explanation might be methodological in nature. The variance in the responses was greater in lower educated than in higher educated participants, among others caused by substantial clustering around the midpoint of the lifeline. Lower educated participants may tend to give more random answers than higher educated participants, who may be better informed about population statistics (Lee & Smith, 2016). This phenomenon is likely to bias associations toward the null (Bruine de Bruin & Carman, 2018). A second, substantial explanation is that higher educated older individuals may be more at ease when considering how much future they have left because they have a greater sense of accomplishment in their lives (Erikson, 1950). Thus, they may be better able to think rationally about where they are in their lifetime and derive this perception more often from their perceived health. In contrast, lower educated individuals likely have experienced more hardship and may more often feel helpless in the face of the future—a feeling that is not necessarily related to health (Mirowsky & Ross, 2000). Future research should further address the differences between lower and higher educated individuals in perceptions of their lifetime.

We furthermore observed a moderately high association between the numerical SA and SLE measures, whereas in studies that reported associations between SA and SLE, this association was much weaker. An explanation for this discrepancy may be that the samples in the studies cited had a broader age range and a younger mean age (between 58 and 65 years) than our own sample (mean age: 74 years). Possibly, for younger people, their remaining SLE is harder to estimate than for older people irrespective of their subjective age, which would have a dampening effect on the SA-SLE association. This is an issue for further research.

One limitation of this study is that we cannot distinguish if subjective lifetime precedes health status or vice versa. In the first case, it might be possible to influence the perception of lifetime such that health would improve, as has been shown already by a few experimental studies (e.g., Stephan et al., 2013). In the other case, subjective lifetime may mediate the association between health and survival time. It was not the purpose of our study to establish causality, and this issue awaits future research. Another, obvious limitation of this study is the high percentage of nonresponders to the numerical question about SLE. Nonresponse percentages such as in our study, however, are not uncommon (Mirowsky, 1999; Shmotkin, 1992), also in studies using a different operational definition such as probability to reach a certain age (Teppa et al., 2015). In a study comparing a future-oriented Cantril ladder and questions about future expectations, Staats et al. (1993) pointed out that nonresponse was higher than 20% on the concrete questions but only 1% on the Cantril ladder. Like in our own study, nonresponse was higher in the lower educated. These authors forward as one explanation the difficulty of the task to specify expectations about the future. In-depth cognitive interviews may help to understand the reasons for specific responses or for not responding (Lee & Smith, 2016). Third, we measure SA and SLE using single questions. A problem using single questions is that their internal consistency cannot be estimated, such as for multi-item scales. However, in our study, we can resort to test–retest correlations, making use of those participants who had two subsequent observations. We found rather high test–retest correlations (.70 for SA, .58 for SLE, and .59 for the lifeline), implying that as the sample members aged 3 or 4 years, their subjective lifetime also “aged” 3 or 4 years. These high correlations correspond to the finding by Rubin and Berntsen (2006) that after the age of 40, people of all age-groups feel a similar amount of years younger than their actual age. A fourth issue is our implicit assumption that the graphical and numerical measures actually measure the same concepts. Our findings give rise to the possibility that this is not the case. Further research, again involving cognitive interviewing, may help clarify this issue.

## Conclusions

Our study combines two subjective lifetime perspectives that so far have been studied in largely separate research traditions: subjective age and subjective life expectancy. As a recent review states, integrating both perspectives and extending methodological procedures will inform a “lifespan theory of subjective time” and deepen our understanding of human aging (Gabrian et al., 2017). Although our study confirms that older adults’ subjective lifetime perspectives are associated with health, all of the associations were rather weak. This suggests that other factors are more important in shaping each perspective, such as optimism, living arrangements, and memory functioning (Palgi et al., 2019). Furthermore, based on our results, we cannot conclude that health is more closely linked to one perspective over another. Such a conclusion would require that both subjective lifetime perspectives can be measured equally well, and our study shows that both graphical and numerical measures involve their own problems, in particular regarding measurement of SLE. Measurement of SA seemed less problematic, yet the graphical and numerical measures showed contrasting associations with self-rated health. What we can conclude is that depressed affect seems more closely linked to SA than to SLE in both operational definitions.

In all, many caveats remain in studying subjective lifetime perspectives, with perhaps the most urgent one the choice of the measurement instrument. Researchers in the field are advised to think very thoroughly on what measures they want to use in order to study their research questions. Or, if they use already collected data, to think thoroughly about what limitations these have for addressing specific research questions.

## Supplemental Material

sj-pdf-1-jah-10.1177_08982643211004001 – Supplemental Material for Capturing Subjective Age, Subjective Life Expectancy, and Their Links With Older Adults’ Health: The Dutch Longitudinal Aging Study Amsterdam Click here for additional data file. Supplemental Material, sj-pdf-1-jah-10.1177_08982643211004001 for Capturing Subjective Age, Subjective Life Expectancy, and Their Links With Older Adults’ Health: The Dutch Longitudinal Aging Study Amsterdam by Dorly J. H. Deeg, Erik J. Timmermans and Almar A. L. Kok in Journal of Aging and Health
