# Supplementary material for: Capturing Subjective Age, Subjective Life Expectancy, and Their Links With Older Adults’ Health: The Dutch Longitudinal Aging Study Amsterdam
Source: J Aging Health. 2021 Mar 31;33(7-8):633–44. doi: 10.1177/08982643211004001 (PMC8236662; doi:10.1177/08982643211004001)
Supplement: sj-pdf-1-jah-10.1177_08982643211004001 – Supplemental Material for Capturing Subjective Age, Subjective Life Expectancy, and Their Links With Older Adults’ Health: The Dutch Longitudinal Aging Study Amsterdam [file sj-pdf-1-jah-10.1177_08982643211004001.pdf]

Supplement: Capturing subjective age, subjective life expectancy, and their links with older adults' health: the Dutch Longitudinal Aging Study Amsterdam

Table S1. Associations of subjective age with health, for different definitions of subjective age

Table S2. Sensitivity analyses with subjective lifetime measures from which 'unrealistic' values are omitted. Associations of subjective age (N-SA) and subjective life expectancy (LL-SLE, N-SLE) with health

Table S3. Sensitivity analyses with subjective lifetime measures from which 'unrealistic' values are omitted. Associations with health: estimates when both N-SA and N-SLE are included in one model

Table S1. Associations of subjective age with health, for different definitions of subjective age

	Physical performance			Self-rated health			Depressive symptoms		
	Model 1	Model 2	Model 3	Model 1	Model 2	Model 3	Model 1	Model 2	Model 3
Subjective age lifeline (LL-SA)	+0.002 (p=0.475) Wald=0.510	-0.002 (p=0.475) Wald=0.510	-0.107 (p=0.603) Wald=0.271	+0.007 (p<0.001) Wald=17.819	-0.007 (p<0.001) Wald=17.819	-0.529 (p<0.001) Wald=17.731	+0.003 (p=0.027) Wald=4.892	-0.003 (p=0.027) Wald=4.892	-0.262 (p=0.024) Wald=5.124
Age	-0.121 (0.004)	-0.119 (0.004)	-0.120 (0.004)	0.013 (0.003)	0.020 (0.002)	0.019 (0.002)	0.021 (0.003)	0.024 (0.002)	0.024 (0.002)
Subjective age number (N-SA)	-0.011 (p<0.001) Wald=13.868	+0.011 (p=0.001) Wald=13.868	+0.774 (p<0.001) Wald=12.655	-0.011 (p<0.001) Wald=27.708	-0.011 (p<0.001) Wald=27.708	-0.815 (p<0.001) Wald=28.718	+0.011 (p<0.001) Wald=49.001	-0.011 (p<0.001) Wald=49.001	-0.810 (p<0.001) Wald=48.228
Age	-0.110 (0.005)	-0.121 (0.004)	-0.120 (0.004)	0.008 (0.003)	0.019 (0.002)	0.018 (0.002)	0.012 (0.003)	0.023 (0.002)	0.021 (0.002)

Model 1: The data in each cell are derived from a GEE model with adjustment for age, sex, and time: subjective age as is

Model 2: The data in each cell are derived from a GEE model with same adjustment: difference of chronological and subjective age

Model 3: The data in each cell are derived from a GEE model with same adjustment: proportional difference of chronological and subjective age (age in denominator)

Table S2. Sensitivity analyses with subjective lifetime measures from which 'unrealistic' values are omitted. Associations of subjective age (N-SA) and subjective life expectancy (LL-SLE, N-SLE) with health*

	Physical performance	Self-rated health	Depressive symptoms
	Model 2	Model 2	Model 2
Subjective age, number (N-SA)	-0.012 (p<0.001) Wald=16.087	+0.012 (p<0.001) Wald=29.233	+0.012 (p<0.001) Wald=51.492
Subjective life expectancy, lifeline (LL-SLE)	+0.004 (p=0.037) Wald=4.332	-0.006 (p<0.001) Wald=25.010	-0.004 (p=0.001) Wald=10.397
Subjective life expectancy, number (N-SLE)	+0.019 (p<0.001) Wald=14.871	-0.032 (p<0.001) Wald=81.534	-0.022 (p<0.001) Wald=51.167

* The data in each cell are derived from a GEE model with adjustment for age, sex, time, and education

Note: For N-SA, values <17 omitted (n=9), for N-SLE and for LL-SLE, values >150 omitted (n=1 and 111, respectively)

Table S3. Sensitivity analyses with subjective lifetime measures from which 'unrealistic' values are omitted. Associations with health: estimates when both N-SA and N-SLE are included in one model

	Physical performance	Self-rated health	Depressive symptoms
	Model 2	Model 2	Model 2
Subjective age number (N-SA)	-0.008 (p=0.012) Wald=5.945	+0.010 (p<0.001) Wald=18.221	+0.011 (p<0.001) Wald=35.555
Subjective life expectancy (N-SLE)	+0.016 (p=0.001) Wald=11.376	-0.030 (p<0.001) Wald=66.739	-0.020 (p<0.001) Wald=40.410

* The data in each cell are derived from a GEE model with adjustment for age, sex, time, and education

Note: For N-SA, values <17 omitted (n=9) and for N-SLE, values >150 omitted (n=1)
